# A Three-Stage Birandom Program for Unit Commitment with Wind Power Uncertainty

**DOI:** 10.1155/2014/583157

**Published:** 2014-05-29

**Authors:** Na Zhang, Weidong Li, Rao Liu, Quan Lv, Liang Sun

**Affiliations:** ^1^School of Electrical Engineering, Dalian University of Technology, Dalian 116024, China; ^2^State Grid Shenyang Electric Power Supply Company, Shenyang 110021, China

## Abstract

The integration of large-scale wind power adds a significant uncertainty to power system planning and operating. The wind forecast error is decreased with the forecast horizon, particularly when it is from one day to several hours ahead. Integrating intraday unit commitment (UC) adjustment process based on updated ultra-short term wind forecast information is one way to improve the dispatching results. A novel three-stage UC decision method, in which the day-ahead UC decisions are determined in the first stage, the intraday UC adjustment decisions of subfast start units are determined in the second stage, and the UC decisions of fast-start units and dispatching decisions are determined in the third stage is presented. Accordingly, a three-stage birandom UC model is presented, in which the intraday hours-ahead forecasted wind power is formulated as a birandom variable, and the intraday UC adjustment event is formulated as a birandom event. The equilibrium chance constraint is employed to ensure the reliability requirement. A birandom simulation based hybrid genetic algorithm is designed to solve the proposed model. Some computational results indicate that the proposed model provides UC decisions with lower expected total costs.

## 1. Introduction

With growing concern about energy and the environment, wind power as the most visible renewable energy generation has been vigorously developed across the world. However, the growth of integrated wind power also brings many dispatching and control challenges due to the nature of wind energy and the characteristics of wind plants. High wind power uncertainties due to unsatisfactory forecast accuracy usually generate additional integration costs, which may be reduced by employing adaptive scheduling policies and developing more appropriate models. Unit commitment (UC) has always been regarded as one of the most crucial processes in system scheduling and the crucial basis for dispatching. How to address the wind power uncertainty in unit commitment has attracted much more concern in recent decades.

Increasing the system reserve level explicitly had been presented when wind uncertainty addressing problem appeared. Soder presented a methodology to estimate the requirements of kinds of reserves (including instantaneous reserve, fast reserve, and slow reserve) by linking with the predefined probability of too low frequency and predefined rate of load shedding in [1], considering multiple uncertainties including wind power. Doherty and O'Malley proposed a further approach in which the amount of SR requirements in each hour was calculated by linking with the reliability targets evaluated by predefined LSI over the year [2]. Ortega-Vazquez and Kirschen proposed a programming method to determine optimal SR requirement which minimizes the expected cost including interruptions and the operating costs [3], in which an uncommon conclusion was drawn that an increased wind power penetration did not necessarily require larger amounts of SR; however, the system reliability level might be compromised correspondingly.

Uncertain programming has been more widely applied in UC modeling to cope with high wind power uncertainties. In particular, stochastic UC (SUC) has been widely studied and applied [4, 5]. The benefit of using stochastic model was evaluated in [6, 7]. Regarding the particular feature of UC problem and its tight coupling relationship with following dispatching process, the two-stage UC model, determining the UC decisions in the first stage and the dispatching decisions in the second stage, is very suitable to cope with wind uncertainties [8]. To ensure the system reliability or the utilization of wind power meeting the required level, or for other aims, chance constraints are also usually applied in SUC modeling [9–11]. Especially in [12], a chance-constrained two-stage stochastic program was formulated, aiming at ensuring a large portion of the wind power output at each operating hour being utilized with a high probability on the premise of maintaining the system reliability in case of sudden fluctuation in wind power output. The two-stage robust UC model, aiming at obtaining the UC decisions which could deal with the anticipated worst scenario, was studied in [13, 14]. Besides stochastic model, fuzzy optimization supplies another approach for UC modeling based on the human's lack of knowledge on exact wind forecast errors [15].

Besides applying advanced modeling methods, much valuable work has been conducted on building adaptive UC policies to cope with wind uncertainty. By using any of the kinds of forecast methods, the wind power forecast accuracy is increased with the forecast horizon shortening, which shows a clear trend when it is from day ahead to several hours ahead. Some studies on UC policy and modeling have been conducted based on this feature, and some positive effects have been shown up. Tuohy et al. [7, 16] presented a rolling UC strategy which committed the system more frequently, and the cost-saving benefit can be obtained by adjusting the UC decisions in intraday scheduling at a certain frequency using the updated wind power data. This offers a proper and practical approach to handle wind power uncertainty in the power systems with intraday electric market. However, in some power systems without electric market, it seems challenging to apply.

Integrating intraday adjustment event into day-ahead UC calculation as well as obtaining a UC decision with the lowest total costs including adjustment cost is another method. In [17], the intraday operation decisions including fast start UC and switching operation modes of combined cycle gas turbines were integrated into the day-ahead scheduling process, by relaxing some of the nonanticipativity constraints based on the two-stage stochastic UC model. In this approach, only the commitment states of fast start units and combined cycle gas turbines can be adjusted in the intraday UC adjustment action. However, such type of units may not be sufficient in most power systems. It means the benefit of integrating intraday adjusting action is limited. Nonetheless, there are usually sufficient conventional thermal units which can start up in 2–6 hours. This type of units is called subfast start unit in this paper. Integrating hours-ahead intraday UC to adjust the commitment states of subfast start units when necessary could also greatly benefit the system reliability and economy, because of the higher accuracy of hours-ahead wind forecast compared with day-ahead forecast. In this regard, a novel three-stage UC, in which a middle stage that determines the intraday hours-ahead UC adjustment decisions is added into the traditional two-stage UC, is developed. The objective of the three-stage UC is to minimize the expected total costs including the expected operation costs as well as the expected startup/shutdown costs according to the final UC decisions, on the premise of satisfying a predefined reliability requirement. It should be noted that in our approach the intraday UC adjustment action is carried out only when the SR capacity is not sufficient according to updated operation and wind forecast data.

The largest challenge of modeling the three-stage UC described above is how to formulate the unrealized next-day hours-ahead forecasted wind power output, which would be used as an input for the intraday UC adjustment stage. When making day-ahead scheduling decisions, the available information about wind power output is its day-ahead forecast value and the statistical forecast error or an uncertain wind forecast result which includes both. The only available information about intraday forecasted wind power in day-ahead scheduling is the statistical forecast error. It can be found that the day-ahead forecasted wind power and intraday hours-ahead forecasted wind power, which are seemingly unable to connect with each other, could build up mathematical relationship based on the possible actual wind power as a medium. The actual wind power output can be formulated as a random variable according to day-ahead forecast data. Considering the random characteristic of intraday wind power forecast errors, the intraday hours-ahead forecasted wind power could also be expressed as a random variable, which takes the value of the stochastic actual wind power output with stochastic hours-ahead forecast errors. It means that the intraday hours-ahead forecasted wind power and intraday UC adjustment event are both with twofold randomness when being considered in day-ahead scheduling. There do exist many scenes of such twofold phenomena in practical situations. In order to build mathematical models that are closer to such actual situations, a so-called birandom variable was proposed by operations research scholars [18]. The birandom variable and birandom programming have been successfully applied to various uncertain programming problems including the vendors selection problem [19], the flow shop scheduling problem [20], the inventory problem [21], and the hydropower station operation [22]. In this paper, the birandom programming is applied to our proposed three-stage UC modeling with wind power uncertainties. To formulate the reliability constraints, chance measures are used in programming formulation.

To solve the UC problems, many methods have been studied, including Lagrangian relaxation algorithm (LR) [23], dynamic programming method (DP) [24], mixed integer programming method (MIP) [25], benders decomposition method [26], genetic algorithm (GA) [27, 28], and some other algorithms. GA is a kind of stochastic search method for optimization problems based on the mechanics of natural selection and natural genetics. It has been successfully used in providing optimal or near optimal solutions to many sophisticated optimization problems. Especially, GA is very effective to solve mathematical programming in an uncertain environment. In [18, 19, 21], the birandom programming problems all were solved by standard or hybrid GA method and obtained satisfactory results. In this paper, a birandom simulation based hybrid GA is designed to solve the proposed three-stage UC model. And to speed up the computation, MILP method is applied to solve each UCED subproblem.

In this paper, an equilibrium chance-constrained three-stage birandom programming UC (ECTBUC) model which integrates expected stochastic intraday UC adjustment event is proposed. The remainder of this paper is organized as follows.  Section 2 presents the UC policy and the mathematical formulation of the proposed model.  Section 3 designs a birandom simulation based hybrid intelligent algorithm for solving the presented model. In Section 4, some numerical studies are provided to illustrate the effectiveness of the model. Finally, some conclusions of this work are given in Section 5.

## 2. Mathematical Formulation

### 2.1. UC Policy

**Figure 1:**
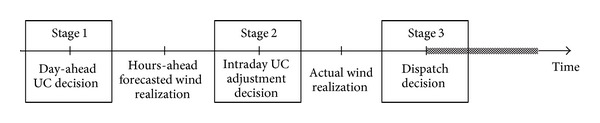
Timeline of proposed three-stage decision method.

A three-stage decision method is employed for the proposed UC problem. An additional middle stage is contained to modify UC schedule of subfast start units several hours earlier when the reliability requirement cannot be satisfied, estimated using the updated wind realization and the hours-ahead wind power forecasting. Therefore, in the UC policy proposed in this paper, the first stage is making day-ahead UC decisions of nonfast start units considering the stochastic intraday UC adjustment event; the second stage is estimating the reliability level according to the ultra-short-term wind power forecasting and making UC adjustment decisions of the subfast start units when the reliability level was estimated unsatisfactory; the third stage is making UC decisions of fast start units and the dispatching decisions. The timeline of the proposed policy is shown in Figure 1.

### 2.2. Intraday Hours-Ahead Forecasted Wind Power Output Uncertainty Modeling

In the policy proposed in this paper, the intraday UC adjustment event should be integrated in the day-ahead UC decision-making process. Due to the fact that the intraday UC adjustment decision should be determined several hours earlier because of the minimum uptime/downtime of subfast start units, the intraday hours-ahead wind power forecasting results should be applied in day-ahead UC modeling. How to model the intraday ultra-short-term forecasted wind power output which cannot be realized when making the day-ahead UC decisions is the key.

As mentioned in Section 1, based on its random nature, wind power forecast error can be regarded as a random variable, following a statistical probability distribution which can be obtained by historical forecast error statistics. Assume that the day-ahead forecasted wind power output during time interval *t* is *w*_*t*_^daf^ and the day-ahead forecast error $\Delta {\tilde{w}}_{t}^{\text{daf}}$ is estimated to follow a probability distribution *F*_*t*_^daf^ according to statistical analyzing; then the actual wind power output, ${\tilde{w}}_{t}=w_{t}^{\text{daf}}+\Delta {\tilde{w}}_{t}^{\text{daf}}$, also is a random variable which can be expressed as ${\tilde{w}}_{t}\sim F_{t}^{\prime \text{haf}}$. Similarly, but in turn, if the hours-ahead forecast error $\Delta {\tilde{w}}_{t}^{\text{haf}}$ is estimated to follow a probability distribution *F*_*t*_^haf^, then, based on each realized actual wind power output *w*_*t*_, the hours-ahead forecasted wind power output ${\tilde{w}}_{t}^{\text{haf}}=w_{t}-\Delta {\tilde{w}}_{t}^{\text{haf}}$ can also be considered as a random variable expressed as ${\tilde{w}}_{t}^{\text{haf}}\sim F_{t}^{\prime \text{haf}}$. As a result, the hours-ahead forecasted wind power output could be formulated as a birandom variable expressed as ${\tilde{\tilde{w}}}_{t}^{\text{haf}}\sim F_{t}^{\prime \text{daf}}({\tilde{w}}_{t})$, ${\tilde{w}}_{t}\sim F_{t}^{\prime \text{daf}}$. (The basic concepts on birandom theory can be found in the Appendix.) For example, assume the day-ahead and hours-ahead wind power forecast errors both follow to normal distributions with mean values equal to 0; then the hours-ahead forecasted wind power output could be formulated as ${\tilde{\tilde{w}}}_{t}^{\text{haf}}\sim N({\tilde{w}}_{t},{\left(\sigma_{t}^{\text{haf}}\right)}^{2})$ and ${\tilde{w}}_{t}\sim N(w_{t}^{\text{daf}},{\left(\sigma_{t}^{\text{daf}}\right)}^{2})$, in which *σ*_*t*_^daf^ indicates the standard deviation of day-ahead wind forecast during time interval *t* and *σ*_*t*_^haf^ indicates the stranded deviation of hours-ahead wind forecast during time interval *t*. How to deal with the wind power uncertainty in practical application will be introduced in detail in Section 3.

### 2.3. UC Mathematical Modeling

In this paper, we develop an equilibrium chance-constrained three-stage birandom unit commitment formulation, which contains an additional middle stage to modify UC schedule of subfast units several hours earlier. In our formulation, the day-ahead UC decisions (*u*_*g*,*t*_^*s*1^, binary variables indicating the status of unit *g* during time interval *t* determined in day-ahead UC) are the first-stage decisions, the intraday UC adjustment decisions (*u*_*g*,*t*_^*s*2^, binary variables indicating the status of subfast start unit *g* during time interval *t* determined in intraday UC adjustment process) are the second-stage decisions, and the economic dispatch decisions (*p*_*g*,*t*_, continuous variables indicating the power output of unit *g* during time interval *t*) are the third-stage decisions. The commitment of fast start units (*u*_*gt*_, binary variables indicating the status of fast start unit *g* during time interval *t*) is decided in the third stage, as suggested in [29, 30].

In our formulation, the reliability constraints are considered in the intraday UC adjustment stage and dispatching stage. To avoid frequent startup/shutdown in intraday operating, only the necessary startup adjustment is permitted, which means no shutdown decisions can be determined in the intraday UC adjustment process, except for a startup adjustment scheduled in the former intraday UC adjustment process. In this model, the only uncertainty considered is the wind power output. The objective function is the expected total cost, which includes the expected startup/shutdown cost according to the final UC decisions modified by intraday UC adjustment event, the expected generation cost, and the expected load shedding cost. The proposed model is formulated as follows:

(1) min⁡∑t∈T ∑g∈GslowSg(ugts1,ug(t−1)s1)+E{∑t∈T ∑g∈GsubfastSgt(ug,ts2,ug(t−1)s2)+E{∑t∈T ∑g∈GFg(pgt)+∑t∈TCL·Udt}},

where *G* is set of all generation units; *G*_subfast_ is subset of nonfast start units that can start up within the time scale of intraday UC adjustment; *G*_slow_ is subset of units that cannot start up within the time scale of intraday UC adjustment so that the UC decisions of which are determined completely in the first stage; *T* is set of all time intervals; *S*_*g*_(·) is startup/shutdown cost function of unit *g*; *F*_*g*_(·) is power production cost function of unit *g*; *CL* is unserved energy price; *p*_*gt*_ is generation output of unit *g* during time interval *t*; and *Ud*_*t*_ is unserved energy during time interval *t*.

In the objective formulation (1), the startup/shutdown cost of subfast start units is included in the objective function as an expected value because the status of subfast start units can be adjusted in the second stage, which is a stochastic event considered in the day-ahead decision. Load shedding is allowed at a high VOLL, in order to assure schedule feasibility.

The first-stage constraints include

(2) $$\begin{array}{c} u_{gt}^{s1}-u_{g\left(t-1\right)}^{s1}-y_{gt}^{s1}\leq 0,\;\forall g\in G,\;\;\forall t\in T, \end{array}$$

(3) $$\begin{array}{c} u_{g(t-1)}^{s1}-u_{gt}^{s1}-z_{gt}^{s1}\leq 0,\;\forall g\in G,\;\;\forall t\in T, \end{array}$$

(4) ugts1−ug(t−1)s1−ugks1≤0, ∀k:1≤k−(t−1)≤MUg,∀g∉Gfast, ∀t∈T,

(5) ug(t−1)s1−ugts1+ugks1≤1, ∀k:1≤k−(t−1)≤MDg,∀g∉Gfast, ∀t∈T,

(6) $$\begin{array}{c} u_{gt}^{s1},y_{gt}^{s1},z_{gt}^{s1}\in \left\{0,1\right\}\;\forall g\notin G_{\text{fast}},\;\;\forall t\in T, \end{array}$$

where *G*_fast_ is subset of fast start units; MU_*g*_ is minimum uptimes of unit *g*; MD_*g*_ is minimum downtimes of unit *g*; *y*_*gt*_^*s*1^ is startup flag of unit *g* at time interval *t* determined in the first-stage decision; and *z*_*gt*_^*s*1^ is shutdown flag of unit *g* at time interval *t* determined in the first-stage decision.

To obtain more economic and flexible UC decisions, only some necessary startup/shutdown constraints for nonfast start units are considered in the first stage. Startup and shutdown flags are summarized in (2) and (3), respectively. Minimum uptime and downtime constraints for nonfast start units are summarized in (4) and (5), respectively.

The second-stage constraints include

(7) 0≤Che{∑g∈Gugt·p−gt+w~~thaf≥dt+Rt}−α+vt≤1,∀t∈T,

(8) $$\begin{array}{c} u_{gt}^{s2}-u_{g\left(t-1\right)}^{s2}-y_{gt}^{s2}\leq 0,\;\forall g\in G_{\text{subfast}},\;\;\forall t\in T, \end{array}$$

(9) $$\begin{array}{c} u_{g(t-1)}^{s2}-u_{gt}^{s2}-z_{gt}^{s2}\leq 0,\;\forall g\in G_{\text{subfast}},\;\;\forall t\in T, \end{array}$$

(10) ugts2−ug(t−1)s2−ugks2≤0, ∀k:1≤k−(t−1)≤MUg,∀g∈Gsubfast, ∀t∈T,

(11) ug(t−1)s2−ugts2+ugks2≤1, ∀k:1≤k−(t−1)≤MDg,∀g∈Gsubfast, ∀t∈T,

(12) $$\begin{array}{c} y_{gt}^{s2}\leq v_{t}+y_{gt}^{s1},\;\forall g\in G_{\text{subfast}},\;\;\forall t\in T, \end{array}$$

(13) $$\begin{array}{c} y_{gt}^{s2}-y_{gt}^{s1}-q_{gt}=0,\;\forall g\in G_{\text{subfast}},\;\;\forall t\in T, \end{array}$$

(14) zgts2−zgts1+ygt′s1≤1, ∀g∈Gsubfast,  ∀t∈T, ∀t≤t′≤t+MDg, t′∈T,

(15) $$\begin{array}{c} u_{gt}^{s2}\geq u_{gt}^{s1},\;\forall g\in G_{\text{subfast}},\;\;\forall t\in T, \end{array}$$

(16) $$\begin{array}{c} u_{gt}^{s2},y_{gt}^{s2},z_{gt}^{s2},v_{t},q_{gt}\in \left\{0,1\right\},\;\forall g\in G_{\text{subfast}},\;\;\forall t\in T, \end{array}$$

where *α* is minimum reliability requirement; *y*_*gt*_^*s*2^ is startup flag of unit *g* at time interval *t* determined in the second-stage decision; *z*_*gt*_^*s*2^ is shutdown flag of unit *g* at time interval *t* determined in the second-stage decision; *v*_*t*_ is adjustment flag indicating the UC decisions during time interval *t* should be adjusted; and *q*_*gt*_ is flag indicating unit *g* is scheduled to start up in the second-stage decision.

Constraints (7) and (12) are built up to ensure subfast start units can be adjusted to start up in the second stage only when the reliability index, estimated using the updated hours-ahead wind forecast data, is lower than the required value. Constraint (13) determines the adjustment flag of each unit. Constraint (14) indicates that if some unit is scheduled to start up in the second stage, it should not shut down if this unit is scheduled to start up in the following MD_*g*_ hours according to the day-ahead UC decisions. To avoid frequent startup/shutdown of subfast start units, constraint (15) is built up to ensure the unit could not turn off if it is on according to the day-ahead decisions. Startup and shutdown flags and minimum uptime/downtime constraints are summarized in (8)–(11). If the commitment state of one unit is off in the remaining intervals according to day-ahead UC, when to shut down is a “wait and see” decision when this unit starts up in the intraday adjustment.

The third-stage constraints include

(17) ∑g∈Gslowugts1·pgt+∑g∈Gsubfastugts2·pgt  +∑g∈Gfastugt·pgt+wt+Udt=dt, ∀t∈T,

(18) $$\begin{array}{c} u_{gt}^{s2}\cdot {\underline{p}}_{g}\leq p_{gt}\leq u_{gt}^{s2}\cdot {\overset{-}{p}}_{g},\;\forall g\in G_{\text{subfast}},\;\;\forall t\in T, \end{array}$$

(19) $$\begin{array}{c} u_{gt}\cdot {\underline{p}}_{g}\leq p_{gt}\leq u_{gt}\cdot {\overset{-}{p}}_{g},\;\forall g\in G_{\text{fast}},\;\;\forall t\in T, \end{array}$$

(20) $$\begin{array}{c} p_{gt}-p_{g(t-1)}\leq \text{ram}{\text{p}}_{g}^{\text{up}},\;\forall g\in G,\;\;\forall t\in T, \end{array}$$

(21) $$\begin{array}{c} p_{g(t-1)}-p_{gt}\leq \text{ram}{\text{p}}_{g}^{\text{down}},\;\forall g\in G,\;\;\forall t\in T, \end{array}$$

(22) $$\begin{array}{c} \text{C}{\text{h}}^{e}\left\{Ud_{t}\leq 0\right\}\geq \alpha ,\;\forall t\in T, \end{array}$$

(23) $$\begin{array}{c} u_{gt},y_{gt},z_{gt}\in \left\{0,1\right\}\;\forall g\in G_{\text{fast}},\;\;\forall t\in T, \end{array}$$

where ${\underline{p}}_{g}$ is minimum output of unit *g*; ${\overset{-}{p}}_{g}$ is maximum output of unit *g*; ramp_*g*_^up^ is ramp-up limit of unit *g*; ramp_*g*_^down^ is ramp-down limit of unit *g*; and *w*_*t*_ is wind power output during time interval *t*.

Constraint (17) expresses the power balance constraint. Load shedding is allowed to assure schedule feasibility. System reliability constraint is summarized in (22), as an equilibrium chance constraint. Other constraints limit the available space, including the maximum and minimum power capacity, as well as ramp rate limits.

## 3. Solution Method

**Figure 2:**
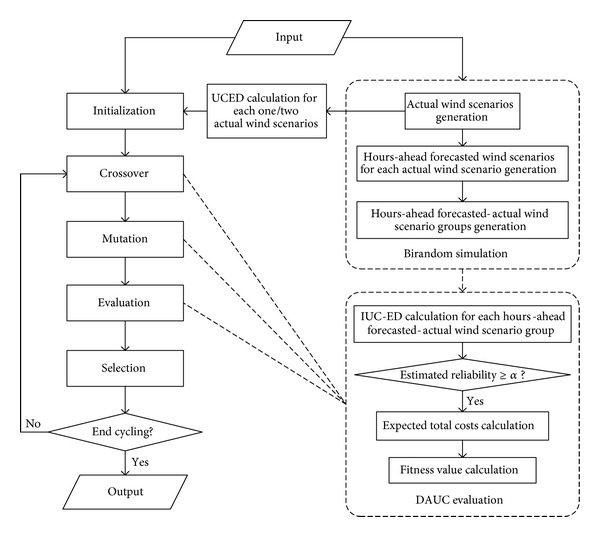
The flowchart of the birandom simulation-based GA algorithm.

GA is a traditional method to solve UC problem and is considered as one of the most effective methods to solve mathematical programming in an uncertain environment, especially in a birandom environment. In [18, 19, 21], the birandom programming problems all were solved by standard or hybrid GA method and obtained satisfactory results. In this regard, GA algorithm is employed to solve the proposed three-stage birandom UC model in this paper. In the proposed model, the employment of birandom variable and birandom programming increases the difficulty of solving. To handle the birandom objective functions and to check the birandom equilibrium chance constraints, a birandom simulation technique, which is similar to stochastic simulation but more complicated, is applied. To speed up the computation, MILP method is used to solve each UCED subproblem. The birandom simulation technique is embedded into a hybrid GA to develop a birandom simulation-based hybrid GA method to solve the proposed CCTSBP UC model. The overall procedure of the applied birandom simulation-based hybrid GA is shown in Figure 2, in which IUC represents intraday UC, and DAUC represents day-ahead UC. The main parts of the algorithm are stated in more detail as follows.

*(a) Wind Scenarios Generation.* Similar to the solving methods of most stochastic UC and birandom programming models, simulation technique is applied. Both the actual wind scenarios and intraday hours-ahead forecasted wind scenarios should be generated as the input of the problem. The wind forecast error distribution feature in the time dimension visibly affects the proposed model. Generating representative actual wind and hours-ahead forecast wind scenarios is the basis for obtaining expected results. Similar to stochastic models, more scenarios can cover much wider uncertainties, however, the computational problems will also show up. In this paper, the initial actual wind scenarios and hours-ahead forecasted wind scenarios are generated using auto regressive moving average (ARMA1,1, as expressed in (24)) approach, according to historical statistic forecast errors on different forecast horizons. And then, simultaneous backward scenario reduction technique is employed to generate the final wind scenarios. The detailed steps are stated as follows. One has

(24) Δwt=αΔwt−1+et+βet−1Δw0=0, e0=0.

1. Identify the parameters, *α*, *β*, and *σ*_*e*_, by solving unconstrained nonlinear optimization problem.
2. Randomly generate *N*_initial_ day-ahead forecast error time series according to (24). Then generate *N*_initial_ actual wind scenarios by day-ahead forecasted wind power output plus forecast errors.
3. Obtain *N*_aw_ actual wind power output scenarios and the probability for each scenario using simultaneous backward scenario reduction technique [31, 32].
4. Generate *N*_haf_ hours-ahead forecasted wind scenarios for each actual wind scenario generated in (3), using the same scenario generation and reduction techniques as generating actual wind scenarios. Then *N*_*s*_ = *N*_haf_ × *N*_aw_ hours-ahead forecasted wind power-actual wind power scenario groups have been generated.

*(b) Hybrid Genetic Algorithm*. In the proposed method, GA is employed to solve the master problem which determines the day-ahead UC decisions, and MILP method is used to solve the subproblem of UCED under each hours-ahead forecasted wind power-actual wind power scenario group. As a result, most of the constraints are considered in solving sub-UCED problem. The minimum on/off hours constraints of conventional units and the reliability constraints are both considered in the master problem solving. The minimum on/off hours of conventional units could be met directly by integer coding in GA algorithm. The reliability constraints are considered by feasibility checking as follows.

(1) *Chromosome Definition.* The chromosomes consist of concatenated positive/negative integers that represent the duration of the “ON/OFF” cycles for each unit during the scheduling period [33]. Positive integers represent durations of ON cycles and negative integers represent duration of OFF cycles of the unit.
(2) *Initialization*. To improve the quality of initial population, parts of initial individuals are generated by solving UCED problems with one or more wind scenarios using MILP method. And to increase the diversity of the population, other parts of initial individuals satisfying the unit minimum uptime and downtime constraints are randomly generated.
(3) *Fitness Function.* Each individual is evaluated by a fitness function as follows:
  (25) $$\begin{array}{c} \text{fitness}=\frac{A}{F}, \end{array}$$
  where *F* represents the expected total costs according to the current UC decision and *A* is a system-dependent constant used to avoid getting too small fitness values.
(4) *Selection*. The selection is based on the roulette-wheel mechanism, so that the chromosome owning a higher fitness value should therefore have a higher chance to be selected.
(5) *Crossover*. To enhance the exchanges of good genes between different chromosomes, a hybrid crossover composed of two crossover operators is used. One operator selects one or two crossing units in the parent chromosomes and exchanges their whole schedules. The other operator randomly selects one or two ON/OFF cycles of randomly selected units and exchanges them and then adjusts the durations of remaining cycles with the least adjusting time to ensure that the sum of durations of selected units is equal to scheduling period.
(6) *Mutation*. Similar to the crossover operators used, a hybrid mutation composed of two mutation operators is applied. One mutation operator changes the whole schedules of selected unit of the parent chromosomes using randomly generated durations of ON/OFF cycles. The other mutation operator changes the cycling duration of one randomly selected position of the selected unit and makes corresponding adjustments to the other ON/OFF cycles of the selected units with the least adjustment times.
(7) *Elitism*. To avoid the destruction through a genetic operator during the evolution process, the best individual of each generation is directly copied to the next.
(8) *Local Search*. To improve the local search ability of the GA algorithm, a local search algorithm proposed in [34] is applied to the best individual at each generation. The main practice of this local search is increasing/decreasing each ON/OFF cycle duration of each unit by 1 and making corresponding adjustment to the other integers with the least adjusting times and then replacing the best individual with the newly generated one, if its fitness value is higher than the original value.

*(c) Feasibility Checking and UC Decision Evaluation*. For each day-ahead UC decision produced by the crossover and mutation operation, the intraday UC adjustment scheduling and economic dispatching should be carried out under each possible hour-ahead forecasted and actual wind scenario group to check their feasibility (the chance constraints) and evaluate their fitness value. This is in practice a birandom simulation. There are two kinds of chance constraints in the proposed model. One is the constraint (7) which ensures the subfast units can be adjusted to start up in intraday operation only when the reliability index is lower than the required value. This chance constraint is considered in the sub-UCED problem under each wind scenario group. The other one is the reliability constraint being considered in GA by feasibility checking. The feasibility checking and evaluation are done according to the following steps.

(1) Set *i* = 0 and *N*′ = 0.
(2) Set *i* = *i* + 1 and *j* = 0.
(3) Set *j* = *j* + 1. Carry out IUC-ED (intraday UC adjustment-economic dispatching) scheduling assuming the hours-ahead forecasted wind scenario *s*_*j*_^haf^-actual wind scenario *s*_*i*_^aw^ group (*s*_*j*_^haf^ − *s*_*i*_^aw^) is realized, and then calculate the total costs *F*_*ij*_ and the total hours satisfying *Ud* ≤ 0  (*T*_*j*_(*Ud* ≤ 0)).
(4) If *j* < *N*_haf_, repeat step (3).
(5) Calculate the expected total costs *F*(*i*) when the scenario *s*_*i*_^aw^ being realized, according to (26)
  (26) $$\begin{array}{c} F\left(i\right)=\overset{N_{\text{haf}}}{\underset{j=1}{\sum }}p_{ij}F_{ij}, \end{array}$$
 where *p*_*ij*_ represents the probability of *s*_*j*_^haf^ if *s*_*i*_^aw^ is realized; *F*_*ij*_ represents the total costs when *s*_*j*_^haf^ − *s*_*i*_^aw^ scenario group is realized.
(6) Calculate Pr{*Ud* ≤ 0} according to (27). If Pr{*Ud* ≤ 0} ≥ *α*, set *N*′ = *N*′ + 1. One has
  (27) $$\begin{array}{c} \text{Pr}\left\{Ud\leq 0\right\}=\frac{\sum_{j=1}^{N_{\text{haf}}}T_{j}\left(Ud\leq 0\right)}{N_{\text{haf}}\times T}. \end{array}$$
(7) If *i* < *N*_aw_, repeat step (2) to step (6).
(8) If *N*′/*N*_aw_ ≥ *α*, return the current UC decision being feasible, and then calculate the expected total costs according to (28), or else the current UC decision is infeasible. Consider
  (28) $$\begin{array}{c} F=\overset{N_{\text{aw}}}{\underset{i=1}{\sum }}p_{i}F\left(i\right), \end{array}$$
 where *p*_*i*_ represents the probability of the *s*_*i*_^aw^ scenario realization; *F* represents the expected total costs according to the current UC decision.

## 4. Numerical Results 

### 4.1. Illustrative Case

**Figure 3:**
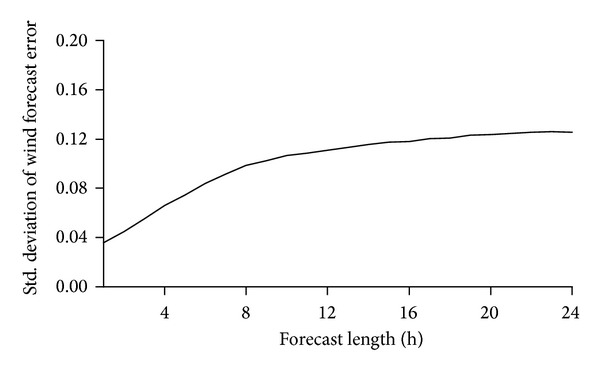
Std. deviation of wind forecast error with different forecast length.

The proposed approach is implemented on a revised 10-unit system presented in [35] first to demonstrate and verify the proposed three-stage UC policy. Units and load data of the 10-unit system are illustrated in Tables 1 and 2, respectively.  Table 3 gives the day-ahead forecast wind power output. The wind forecast errors with different forecast horizons are shown in Figure 3. The intraday UC adjustment stage is assumed to be carried out 4 hours earlier before the real time.

In the genetic algorithm, 10 actual wind scenarios and 5 hours-ahead forecasted wind scenarios for each actual wind scenario are generated. The pop size is 20. The probability of crossover was set as 0.7, and probability of mutation was set as 0.2. The initial individuals include 10 UC decisions obtained at each actual wind scenario, 5 UC decisions obtained at randomly grouped 2 actual wind scenarios, 1 UC result obtained with all the 10 actual wind scenarios, 1 UC result considering the day-ahead forecasted wind as a perfect forecasting, and randomly generated 3 UC schedules meeting the minimum uptimes/downtimes of units. The maximum number of generation is 100. The cost of load shedding is assumed to be 3000$/MWh. To compare with the proposed model, the traditional two-stage stochastic UC was also implemented on the test system.

The UCED and two-stage SUC problems in all case studies have been solved using CPLEX 12 under Matlab. An AMD Core computer at 3.01 GHz and 4 GB of RAM has been used.

Table 4 shows the UC decision according to the traditional two-stage stochastic UC.  Table 5 shows the day-ahead UC decision according to the proposed three-stage birandom UC, in which the red ones present the different states compared with traditional two-stage UC decision. The optimal objective function value using traditional two-stage stochastic UC model is $485760.87, whereas the optimal objective function value using the proposed three-stage birandom UC model is $483832.80. Compared with the traditional two-stage model, it saves approximately $1928 if the proposed approach is used.  Table 6 shows the final UC decision using the proposed approach in wind scenario group 1-1. It can be seen that, during hours 11–14, unit 6 is starting up according to intraday UC adjustment decision, but compared with the UC decision using two-stage stochastic policy, the committed generation capacity is lower, resulting in lower expected total costs. It should be presented that in some wind scenario groups no intraday UC adjustment action should be done. The intraday UC adjustment actions were carried out in 27 simulated scenarios, which were 54 percent of the whole 50 scenarios.

### 4.2. Test Case

**Figure 4:**
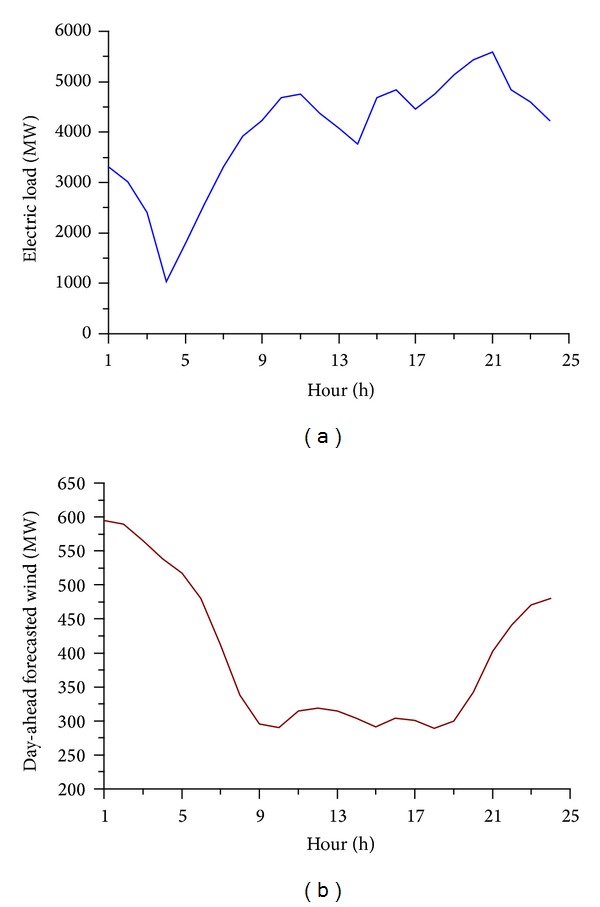
(a) Electric load profile. (b) Day-ahead forecasted wind power output.

The proposed approach is implemented on a revised IEEE118 bus system with 33 conventional units to test its validity. The load and day-ahead forecasted wind power output data are shown in Figure 4. The algorithm parameters setting and the wind forecast error curve are the same as in the illustrative case. In order to analyze the dispatch in real-time scenarios, the resulting schedules were evaluated in 500 randomly simulated wind profile realizations. When applying the proposed policy, the hours-ahead forecasted wind data were also randomly simulated. The expected total costs are summarized at the bottom of Table 7. Results reveal that the application of proposed policy and model reduced operating costs by about 0.4% with respect to traditional two-stage stochastic policy. However, it can be seen that it needed much longer solving time when using proposed model because of simulation method being used. In practice, the computation performance may be improved by using a parallel algorithm, because the fitness calculation and feasibility checking of individuals are independent of each other.

### 4.3. Influence of Forecast Accuracy

**Figure 5:**
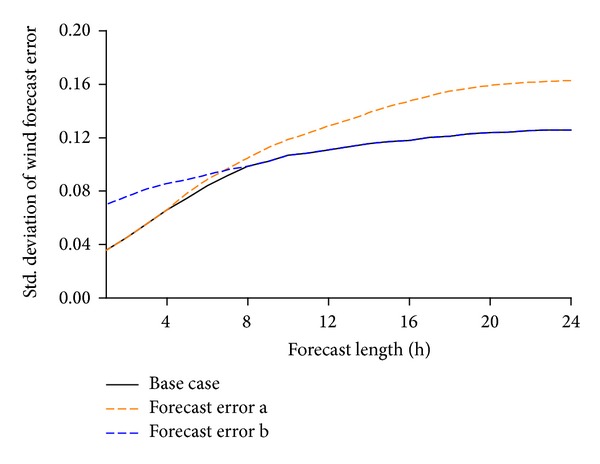
Different wind power forecast error curves for case study.

The main contribution of the proposed UC policy and model is integrating intraday UC adjustment process, on the theoretical basis that the wind forecast accuracy is increased with the forecast length shortening. Another two wind forecast error curves shown in Figure 5 were applied to test the influence of wind forecast accuracy on the cost saving benefit of proposed approach. The two wind forecast error curves both increase the forecast error but in various ways. In forecast error curve a, the ultra-short-term forecast error is the same as the base case; however, when the forecast length is longer than several hours, it gradually increases. In forecast error curve b, the forecast error with longer horizons is the same as the base case, but the ultra-short-term forecast error is higher than that of the base case. 500 randomly simulated wind profile realizations were also used to evaluate the resulting schedules.

Table 8 shows the optimal objective values and the expected total costs of simulation when applying the proposed model and the two-stage stochastic model. It can be seen that the total costs with both simulated wind power forecast error curves and using both UC models are higher than those of base case, correspondingly. Compared with the two-stage stochastic model, the proposed model could reduce the total costs with both two wind power forecast error curves. However, when the wind forecast error follows curve a, the cost savings are higher compared with the base case, whereas when the wind forecast error follows curve b, it saves less. This shows an obvious benefit of higher ultra-short-term wind forecast accuracy if using the proposed model, which means even when the day-ahead forecast accuracy is much lower, the operation costs will not be so high if ultra-short-term forecast accuracy is high, when using the proposed policy and model; however, if the trend of forecast error decreasing with the forecast horizon is not so clear, the cost saving effect of using the proposed model will be reduced.

## 5. Conclusions 

This paper presents a novel three-stage UC policy and a corresponding equilibrium chance-constrained three-stage birandom UC model for the power systems with wind power uncertainty. The main contribution of this paper is to integrate hours-ahead UC adjustment stage into traditional two-stage UC, based on the feature of wind power forecast accuracy that can be increased if the forecast length is shorter. A birandom simulation based hybrid genetic algorithm is designed to solve the proposed model. The following conclusions can be indicated by numerical results.

1. The operation costs of power systems with wind power integration can be reduced by applying the proposed three-stage UC policy and the corresponding birandom UC model.
2. The improvement level of hours-ahead wind power forecast accuracy compared with day-ahead forecast accuracy greatly influences the benefit of applying the proposed UC policy and model. When the day-ahead forecast accuracy is low, the operation costs will not be so high if hours-ahead forecast accuracy is higher, when using the proposed policy and model. However, if the trend of forecast error decreasing with the forecast horizon is not so clear, the cost saving effect of using the proposed model will be reduced.
3. When the day-ahead wind forecast accuracy cannot be satisfactorily improved, improving the ultra-short-term wind forecast accuracy can further reduce the operation costs if using the proposed approach.
4. Solving the proposed birandom UC model needs much longer time. But, in practice, the computation performance may be improved by using a parallel algorithm, because the fitness calculation and feasibility checking of individuals are independent of each other.

## Figures and Tables

**Table 1:** Generator data.

Unit	*p*_max⁡_ (MW)	*p*_min⁡_ (MW)	UT (h)	DT (h)	Ini. State (h)	*a* ($/h)	*b* ($/MWh)	*c* ($/MW^2^h)	Hc ($/h)	Cc ($/h)	*t*_cold (h)	*T*_start (h)
1	455	150	8	8	8	1000	16.19	0.00048	4500	9000	5	8
2	455	150	8	8	8	970	17.26	0.00031	5000	10000	5	8
3	130	20	5	5	−5	700	16.6	0.002	550	1100	4	3
4	130	20	5	5	−5	680	16.5	0.00211	560	1120	4	3
5	162	25	6	6	−6	450	19.7	0.00398	900	1800	4	4
6	80	20	3	3	−3	370	22.26	0.00712	170	340	2	2
7	85	25	3	3	−3	480	27.74	0.00079	260	520	2	2
8	55	10	1	1	−1	660	25.92	0.00413	30	60	0	0
9	55	10	1	1	−1	665	27.27	0.00222	30	60	0	0
10	55	10	1	1	−1	670	27.79	0.00173	30	60	0	0

**Table 2:** Hourly load data.

1–12 h (MW)	700	750	850	950	1000	1100	1150	1200	1300	1400	1450	1500
13–24 h (MW)	1400	1300	1200	1050	1000	1100	1200	1400	1300	1100	900	800

**Table 3:** Day-ahead forecasted wind power output.

1–12 h (MW)	177	171	158	145	142	137	120	109	116	116	117	115
13–24 h (MW)	114	109	107	111	103	90.5	99.6	120	127	137	160	175

**Table 4:** UC decision using traditional two-stage stochastic model.

Unit	Unit state																							
1	1	1	1	1	1	1	1	1	1	1	1	1	1	1	1	1	1	1	1	1	1	1	1	1
2	1	1	1	1	1	1	1	1	1	1	1	1	1	1	1	1	1	1	1	1	1	1	1	1
3	0	0	0	0	0	0	1	1	1	1	1	1	1	1	1	0	0	0	0	0	0	0	0	0
4	0	0	0	0	0	0	1	1	1	1	1	1	1	0	0	0	0	0	1	1	1	1	1	0
5	0	0	0	0	0	1	1	1	1	1	1	1	1	1	1	1	1	1	1	1	1	0	0	0
6	0	0	0	0	0	0	0	0	1	1	1	1	1	1	0	0	0	0	0	0	0	0	0	0
7	0	0	0	0	0	0	0	0	0	0	0	0	0	0	0	0	0	0	0	0	0	0	0	0
8	0	0	0	0	0	0	0	0	0	0	0	0	0	0	0	0	0	0	0	0	0	0	0	0
9	0	0	0	0	0	0	0	0	0	0	0	0	0	0	0	0	0	0	0	0	0	0	0	0
10	0	0	0	0	0	0	0	0	0	0	0	0	0	0	0	0	0	0	0	0	0	0	0	0

**Table 5:** Day-ahead UC decision using proposed model.

Unit	Unit state																							
1	1	1	1	1	1	1	1	1	1	1	1	1	1	1	1	1	1	1	1	1	1	1	1	1
2	1	1	1	1	1	1	1	1	1	1	1	1	1	1	1	1	1	1	1	1	1	1	1	1
3	0	0	0	0	0	0	1	1	1	1	1	1	1	1	1	0	0	0	0	0	0	0	0	0
4	0	0	0	0	0	0	1	1	1	1	1	1	1	0	0	0	0	0	**0**	**0**	**0**	**0**	**0**	0
5	0	0	0	0	0	1	1	1	1	1	1	1	1	1	1	1	1	1	1	1	1	0	0	0
6	0	0	0	0	0	0	0	0	**0**	**0**	**0**	**0**	**0**	**0**	0	0	0	0	0	0	0	0	0	0
7	0	0	0	0	0	0	0	0	0	0	0	0	0	0	0	0	0	0	0	0	0	0	0	0
8	0	0	0	0	0	0	0	0	0	0	0	0	0	0	0	0	0	0	0	0	0	0	0	0
9	0	0	0	0	0	0	0	0	0	0	0	0	0	0	0	0	0	0	0	0	0	0	0	0
10	0	0	0	0	0	0	0	0	0	0	0	0	0	0	0	0	0	0	0	0	0	0	0	0

**Table 6:** The final UC decision using proposed model in wind scenario group 1-1.

Unit	Unit state																							
1	1	1	1	1	1	1	1	1	1	1	1	1	1	1	1	1	1	1	1	1	1	1	1	1
2	1	1	1	1	1	1	1	1	1	1	1	1	1	1	1	1	1	1	1	1	1	1	1	1
3	0	0	0	0	0	0	1	1	1	1	1	1	1	1	1	0	0	0	0	0	0	0	0	0
4	0	0	0	0	0	0	1	1	1	1	1	1	1	0	0	0	0	0	**0**	**0**	**0**	**0**	**0**	0
5	0	0	0	0	0	1	1	1	1	1	1	1	1	1	1	1	1	1	1	1	1	0	0	0
6	0	0	0	0	0	0	0	0	**0**	**0**	*1 *	*1 *	*1 *	*1 *	0	0	0	0	0	0	0	0	0	0
7	0	0	0	0	0	0	0	0	0	0	0	0	0	0	0	0	0	0	0	0	0	0	0	0
8	0	0	0	0	0	0	0	0	0	0	0	0	0	0	0	0	0	0	0	0	0	0	0	0
9	0	0	0	0	0	0	0	0	0	0	0	0	0	0	0	0	0	0	0	0	0	0	0	0
10	0	0	0	0	0	0	0	0	0	0	0	0	0	0	0	0	0	0	0	0	0	0	0	0

**Table 7:** Simulation results of the test case.

	Optimal objective value ($)	Simulated expected total costs ($)	Solution time (sec.)
Two-stage stochastic UC	1202576.93	1202571.29	183
Three-stage birandom UC	1197628.57	1197602.47	8632
Cost savings	4948.36	4968.82	

**Table 8:** Simulation results with different wind power forecast error curves.

	Optimal objective value ($)	Simulated expected total costs ($)
Wind forecast error curve a		
Two-stage stochastic UC	1203659.87	1203638.94
Three-stage birandom UC	1198043.92	1198054.63
Cost savings	5615.95	5584.31
Wind forecast error curve b		
Two-stage stochastic UC	1202987.43	1202996.53
Three-stage birandom UC	1198928.57	1198924.86
Cost savings	4058.86	4071.67

## Appendix

### A. Some Basic Concepts on Birandom Theory

Stochastic programming was set up independently by Dantzig, Charnes, and Cooper in the 1950s. Over the past few decades, stochastic programming modeling and solving have gotten considerable development. It has been widely applied to the real-life decision making problem in many fields such as finance, production scheduling, certainly, and power system planning and dispatching, especially in the system scheduling with large-scale renewable energy power in recent years. To describe the twofold randomness which is ubiquitously throughout the real life, Peng and Liu proposed birandom variable and birandom programming [18]. In the uncertain theory, a birandom variable is a mapping from a probability space to a collection of random variables. Roughly speaking, it is a “random variable” taking random variable values. Some knowledge of birandom theory is introduced in this section [18].

#### A.1. The Definition of Birandom Variable

A birandom variable *ξ* is a mapping from a probability space (*Ω*, *A*, Pr) to a collection *S* of random variables for any Borel subset *B* of the real line *R*. The induced function Pr{*ξ*(*ω*) ∈ *B*} is a measurable function with respect to *ω*.

An* n-*dimensional birandom vector *ξ* is a mapping from the probability space (*Ω*, *A*, Pr) to a collection of* n-*dimensional random vector such that Pr{*ξ*(*ω*) ∈ *B*} is a measurable function with respect to *ω* for any Borel subset *B* of the real space *R* ^*n*^.

#### A.2. Expected Value Operator

The expected value of a birandom variable is somewhat similar to that of a random one.

Let *ξ* be a birandom variable defined on the probability space (*Ω*, *A*, Pr). Then the expected value of birandom variable *ξ* is defined as

(A.1) E[ξ]=∫0∞Pr{ω∈Ω|E[ξ(ω)]≥t}dt−∫0∞Pr{ω∈Ω|E[ξ(ω)]≤t}dt,

provided that at least one of the above two integrals is finite.

#### A.3. Chance Measures

For applying to further mathematical analysis, several methods have been suggested to define the chance measures of birandom event, two of which are introduced in this section.

Let *ξ* = (*ξ*_1_, *ξ*_2_,…, *ξ*_*n*_) be a birandom vector on (*Ω*, *A*, Pr) and *f* : *R* ^*n*^ → *R* ^*m*^ a vector-valued Borel measurable function. Then the primitive chance of birandom event characterized by *f*(*ξ*) ≤ 0 is a function from (0,1] to [0,1], defined as

(A.2) Ch{f(ξ)≤0}(α) =sup⁡{β ∣ Pr{ω∈Ω ∣ Pr{f(ξ(ω))≤0}≥β}≥α}.

The equilibrium chance of birandom event characterized by *f*(*ξ*) ≤ 0 is defined as

(A.3) $$\begin{array}{c} \text{C}{\text{h}}^{e}\left\{f\left(\xi \right)\leq 0\right\}=\underset{\alpha \in \left[0,1\right]}{\sup}\left\{\alpha \;|\;\text{Ch}\left\{f\left(\xi \right)\leq 0\right\}\left(\alpha \right)\geq \alpha \right\}. \end{array}$$

It was proved in [21] that

(A.4) Che{f(ξ)≤0}≥α⟺Pr{ω∈Ω ∣ Pr{g(ξ(ω))≤0}≥α}≥α.

The equilibrium chance of birandom event has been widely applied in the chance-constrained program modeling with birandom parameters in recent years, and some good results have been obtained. The equilibrium chance is employed in the proposed model in this paper to describe the system reliability constraints in birandom environment.
